# Impact of radial compression protocols on radial artery occlusion and hemostasis time in coronary angiography

**DOI:** 10.1007/s12928-022-00896-6

**Published:** 2022-12-07

**Authors:** Sachiko Takamatsu, Nobuyuki Kagiyama, Naohiko Sone, Kiyotaka Tougi, Shuichiro Yamauchi, Takuya Yuri, Nobuhisa Ii, Tomoko Sugimoto, Motomaru Masutani, Atsushi Hirohata

**Affiliations:** 1 Department of Nursing, The Sakakibara Heart Institute of Okayama, Okayama, Japan; 2 Department of Digital Health and Telemedicine R&D, Juntendo University, 2-1-1 Hongo, Bunkyo-Ku, Tokyo, Japan; 3 Department of Cardiovascular Biology and Medicine, Juntendo University, Tokyo, Japan; 4 Cardiovascular Medicine, The Sakakibara Heart Institute of Okayama, Okayama, Japan; 5 Department of Nursing, Hakuhoukai Central Hospital, Hyogo, Japan; 6 Cardiovascular Medicine, Hakuhoukai Central Hospital, Hyogo, Japan

**Keywords:** Catheter angiography, Trans-radial access, Hemostasis, Radial artery occlusion

## Abstract

Protocols for hemostasis after trans-radial approach (TRA) vary depending on the institute as there is no established evidence-based protocol. This study aimed to investigate the clinical implications of radial compression protocols. Consecutive patients who underwent outpatient invasive catheter angiography before and after April 2018 were treated with traditional and new protocols, respectively. Using the same hemostasis band, in the conventional protocol, fixed amount of air was removed soon after the procedure, 2 h later, and 3 h later, whereas the air was removed as much as possible every 30 min in the new protocol. A total of 1842 patients (71 ± 10 years old, 77% male) were included. Compared with the traditional protocol group (*n* = 1001), the new protocol group (*n* = 841) had a significantly lower rate of dual antiplatelet therapy (35% and 24% in the traditional and new groups, respectively, *p* < 0.001). The time required for complete hemostasis was approximately one-third with the new protocol (190 ± 16 and 66 ± 32 min, *p* < 0.001) with no clinically relevant bleeding. The incidence of radial artery occlusion (RAO) was 9.8% and 0.9% in the traditional and new protocol groups, respectively (*p* < 0.001). After adjusting for covariates, the new protocol was associated with a reduced risk of RAO (odds ratio 0.10, *p* < 0.001) and a shorter hemostasis time (odds ratio 0.01, *p* < 0.001). The new protocol for hemostasis after TRA was strongly associated with a shorter hemostasis time and a lower rate of RAO.

## Introduction

The trans-radial approach (TRA) was established over the past three decades. It is now widely accepted in many situations as the standard access for invasive coronary catheter procedures [1–6]. Since the radial arteries are located superficially in the forearm, access is easy, and the method is safe. Unlike trans-femoral catheterization, TRA does not require long-term bed rest which could increase the risk of complications, such as venous thrombosis. Studies have demonstrated that TRA is generally safer than trans-femoral and trans-brachial approaches [7–11].

Radial artery occlusion (RAO) is one of the most common and problematic complications and is reported to occur in 2–11% of cases after TRA [12]. Since the radial artery is smaller in diameter than the femoral and brachial arteries, sheaths sometimes injure the radial artery walls during TRA, resulting in RAO after the procedure [13]. RAO is most often observed as a diminished or nonpalpable pulse and rarely causes numbness or coldness in the hands [14, 15]. Even when the patient is asymptomatic, RAO is a significant problem, as it may limit future access routes for coronary catheters [16]. Many clinical factors including the radial artery diameter, sheath size, and antiplatelet therapies, are thought to be risk factors for RAO [14, 17–20]. Of these, hemostasis time and the procedure itself are more easily modified. However, most hospitals use a commercially recommended protocol that focuses excessively on the risk of bleeding. This may be because an evidence-based optimal radial compression protocol is lacking. In this study, we sought to elucidate the impact of the new protocol on hemostasis time and the incidence of RAO after TRA.

## Methods

**Fig. 1:**
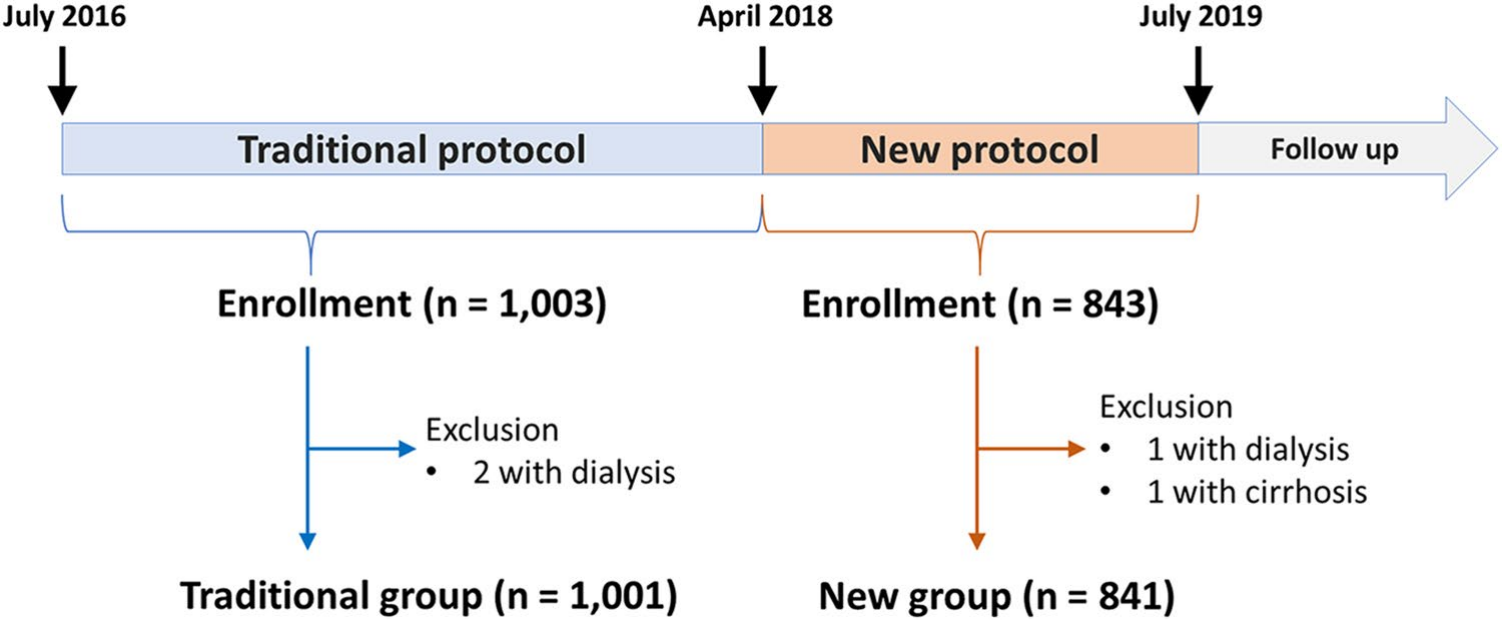
Radial compression protocols. In both protocols, 16 ml of the air is injected and then gradually removed. In the traditional protocol, the amount of the timing of air removal is fixed, whereas the air is removed as possible without bleeding in the new protocol

The present study was a single-center retrospective observational study designed to evaluate the effectiveness of a new hemostasis protocol for trans-radial invasive cardiac catheterization. Consecutive patients who underwent invasive trans-radial coronary angiography at our outpatient clinic between April 2018 and July 2019 were treated with the new hemostasis protocol. This protocol was fixed based on our pilot experience. The data were compared with that of the consecutive patients who were treated with the traditional hemostasis protocol between July 2016 and March 2018. The inclusion criteria were (1) outpatients who underwent trans-radial coronary angiography in the Department of Cardiology and (2) adult patients (≥ 20 years old). In addition, (1) patients who underwent catheter intervention, (2) patients with acute coronary syndrome, and (3) patients with hemodialysis or cirrhosis, were excluded. Coronary angiography was performed by an experienced cardiologist using a TRA. After inserting a 4- or 5-Fr sheath (Radifocus Introducer IIH, Terumo Corporation, Tokyo, Japan), 50 U/kg of heparin was injected intravenously. Immediately after the procedure, the sheath was removed. Subsequently, hemostasis was achieved using the traditional commercially recommended protocol before April 2018, after which the new protocol was used. We used a commercially available hemostasis band (TR band; Terumo Corporation, Tokyo, Japan) in both protocols. In the traditional protocol, after initially injecting 16 mL of air into the band, patients were transferred to a postoperative monitoring room, where 2 mL of air was immediately removed. Then, 4 and 10 mL of air was removed 2 and 1 h later, respectively (Fig. 1, upper panel). If the puncture site bled, half volume of air was added. Once the air has been completely removed, the band was detached and hemostasis was completed. In the new protocol, after initially injecting 16 mL of air, as much of the air was removed as possible without bleeding, immediately after the patient was transferred to the recovery room. The same removal procedure was repeated every 30 min until the air was completely removed (Fig. 1, bottom panel). Performing physicians were classified as follows: Residents (who have < 3 years of experience in transradial catheter angiography), Fellows (who have 3–6 years of experience), and Attendings (board-certified cardiologists who have more than 6 years of experience). Written informed consent was waivered given the retrospective non-invasive observational nature of the study under the Ethical Guidelines for Medical and Health Research Involving Human Subjects issued by the Japanese Ministry of Health, Labor and Welfare. The study protocols complied with the guidelines of the Declaration of Helsinki and were approved by the Ethics Committee of Sakakibara Heart Institute of Okayama.

### Outcome measures

The incidence of RAO was tracked as the primary efficacy endpoints, and the time to achieve hemostasis was the co-primary endpoint. The rate of clinically relevant bleeding defined as bleeding requiring surgical treatment, blood transfusion, or unexpected hospital stay was counted as the safety endpoint. The time required to achieve hemostasis was measured from the time when the sheath was removed to the time when the band was detached. Hemostasis time was considered to be prolonged if it exceeds 180 min. RAO was assessed 6 months after the procedure by physical examination and/or ultrasound examination by an experienced cardiologist at outpatient clinics or operation room where follow-up angiography was performed. If the physician did not feel pulse after careful examination, did not confirm blood flow by color flow Doppler, or was not able to puncture the artery, the artery was considered to be occluded. Ultrasound was optional and not routinely performed. Clinically relevant bleeding was defined as a bleeding event that required surgical treatment, blood transfusion, or hospital admission.

### Statistical analysis

The data are presented as the mean ± standard deviation for continuous variables and as frequency (%) for categorical variables. Group differences were evaluated using the Student’s *t* test for continuous variables and the Fisher’s exact tests for categorical variables. Logistic regression models were applied to assess the effectiveness of the new protocol after adjusting for confounders. All statistical analyses were performed using R (version 4.0.3, Vienna, Austria). Two-sided *p* value < 0.05 was considered significant in all analyses.

### Data availability

The data that support the findings of this study are available from the corresponding author upon reasonable request.

## Results

### Population

**Fig. 2:**
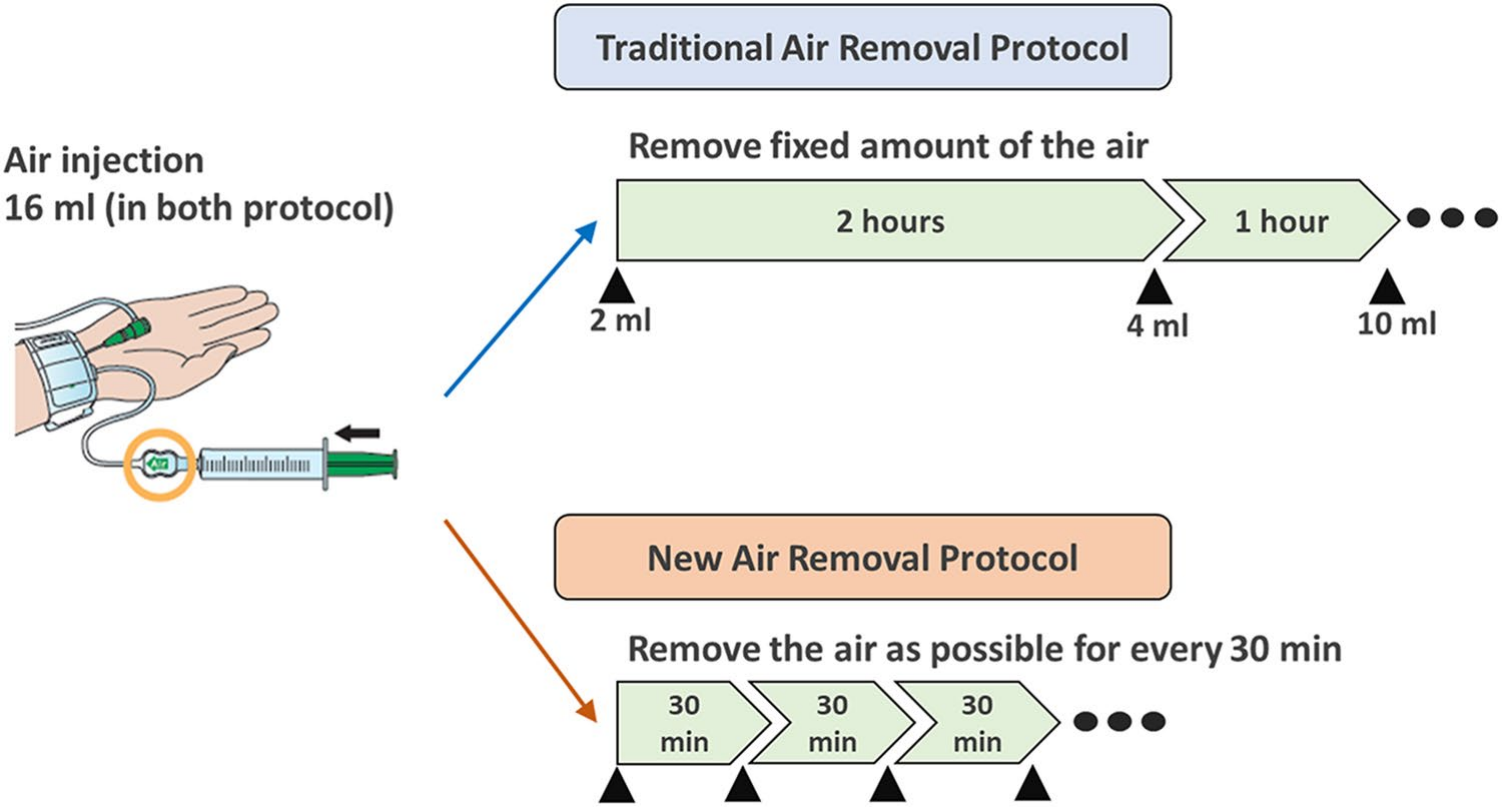
Patient enrollment chart. In total, 1001 and 841 patients were enrolled in the analysis

A total of 1846 patients underwent trans-radial coronary angiography. After excluding three patients who required hemodialysis and one patient with cirrhosis, 1842 patients (1001 and 841 in the traditional and new protocol groups, respectively) were finally included in the analysis (Fig. 2). Patient characteristics are summarized in Table 1. Age, sex, systolic blood pressure, body surface area, body mass index, creatinine level, and platelet count were not significantly different between the two groups. The diastolic blood pressure was slightly lower in the new protocol group. A larger sheath was used more frequently in the new group. The prevalence of hypertension and dual antiplatelet therapy was also higher in the new group. The operator’s experience was not different between the groups.

**Table 1:** Patient characteristics

	Traditional protocol *N* = 1001	New protocol *N* = 841	*p* value
Age, years	71 ± 10	71 ± 10	0.20
Male	756 (76%)	660 (78%)	0.15
Systolic BP, mmHg	131 ± 20	130 ± 18	0.30
Diastolic BP, mmHg	74 ± 12	72 ± 12	0.021
BMI, kg/m^2^	24.6 ± 5.4	24.6 ± 3.5	0.80
Body surface area, m^2^	1.67 ± 0.20	1.69 ± 0.18	0.059
Creatinine, mg/dL	0.90 ± 0.36	0.92 ± 0.19	0.30
Platelet, /mm^3^	19.7 ± 7.7	20.2 ± 5.8	0.12
Sheath size, Fr			< 0.001
4	966 (97%)	767 (91%)	
≥ 5	35 (3.5%)	74 (8.8%)	
Previous cath, times	3.78 ± 2.99	3.33 ± 2.99	0.001
Hypertension	721 (72%)	513 (61%)	< 0.001
Diabetes	388 (39%)	294 (35%)	0.10
SAPT	378 (38%)	330 (39%)	0.50
DAPT	347 (35%)	206 (24%)	< 0.001
DOAC	56 (5.6%)	34 (4.0%)	0.13
Warfarin	43 (4.3%)	23 (2.7%)	0.079
Operator			0.60
Residents	228 (17%)	94 (18%)	
Fellows	535 (41%)	225 (43%)	
Attendings	552 (42%)	208 (39%)	

### Primary endpoint: radial artery occlusion

**Fig. 3:**
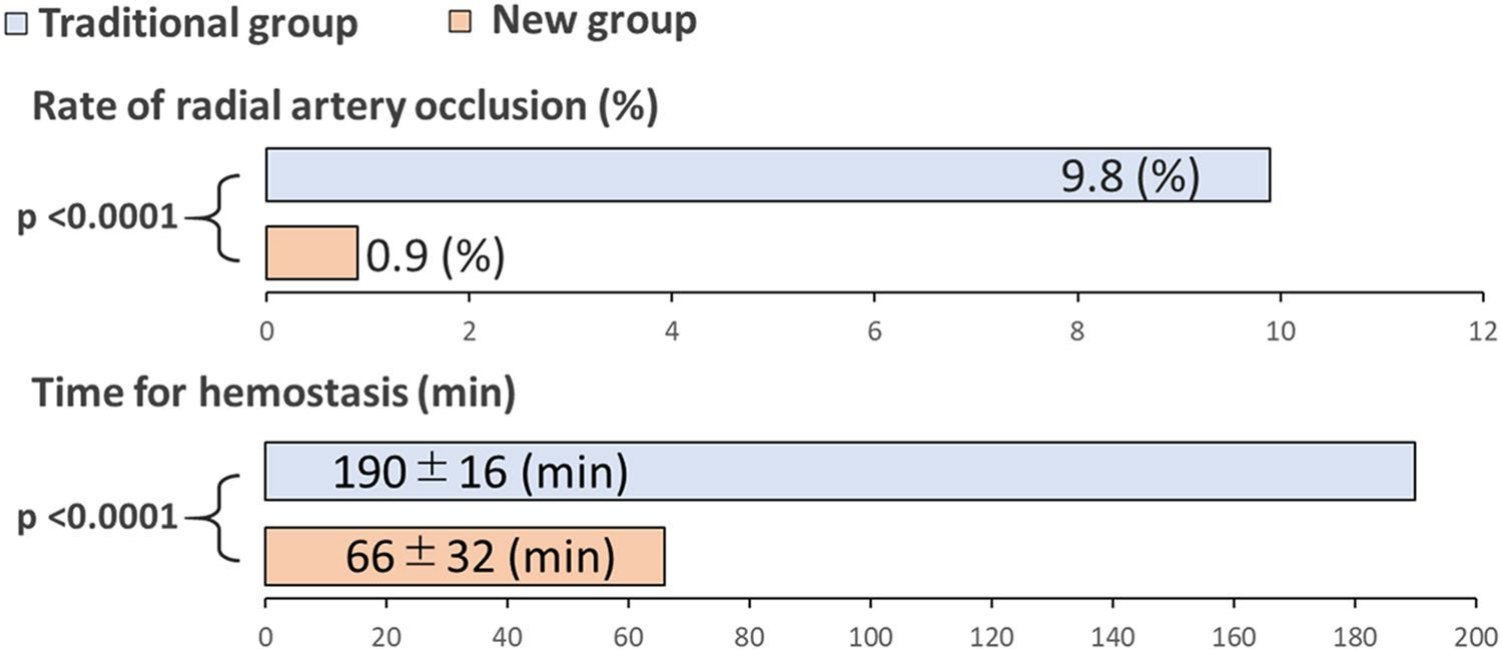
Radial compression time and the rate of radial artery occlusion. The new protocol group had dramatically shorter time for complete hemostasis and a lower rate of radial artery occlusion

A 6-month follow-up was available for 1315 patients (71.4%). The incidence of RAO was less than one-tenth. RAO was detected in 64 (9.9%) with the traditional protocol and 6 (0.9%) with the new protocol (Fig. 3, upper panel). We also constructed multivariate logistic regression models to assess the association between RAO and its risk factors. Since the protocol and hemostasis were very closely correlated as stated below, we created two models: one with the new protocol and the other with time to hemostasis. The model showed that both the new protocol (OR 0.09, *p* < 0.0001) and time of hemostasis (OR 1.18/10 min, *p* < 0.0001) were significantly associated with RAO (Table 2). The number of previous trans-radial catheterization and body surface area were also significantly associated with RAO in both models.

**Table 2:** Risk for radial artery occlusion

	Odds ratio	95% CI	*p* value
New protocol	0.10	0.04–0.23	< 0.001
Hypertension	0.55	0.32–0.96	0.03
Previous cath times	1.17	1.10–1.25	< 0.001
SAPT	1.42	0.65–3.08	0.38
DAPT	1.54	0.69–3.43	0.29
Body surface area	0.09	0.02–0.35	< 0.001
Operator: fellows (vs. residents)	1.50	0.67–3.35	0.33
Operator: attendings (vs. residents)	1.34	0.60–2.98	0.48

	Odds ratio	95% CI	*p* value
Time of hemostasis/10 min	1.18	1.11–1.26	< 0.001
Hypertension	0.58	0.34–1.00	0.04
Previous cath times	1.16	1.09–1.24	< 0.001
SAPT	1.33	0.61–2.90	0.47
DAPT	1.44	0.65–3.21	0.37
Body surface area	0.08	0.02–0.35	< 0.001
Operator: fellows (vs. residents)	1.65	0.74–3.70	0.22
Operator: attendings (vs. residents)	1.50	0.68–3.35	0.32

### Co-primary endpoint: time to complete hemostasis

The time to achieve hemostasis was significantly shorter in the new protocol. It was approximately one-third of that in the traditional protocol (190 ± 16 min in the traditional protocol vs. 66 ± 32 min in the new protocol, *p* < 0.001; Fig. 3, lower panel). Complete hemostasis was achieved at the first check at 120 min after the air inflation in the majority of patients in the traditional protocol group, suggesting that the actual time required for the hemostasis might be shorter than 120 min in these patients. Prolonged hemostasis time (> 180 min) was required for 294 (29%) and 4 (0.5%) patients in the traditional and new protocols, respectively. None of the patients in either group experienced clinically relevant bleeding that required surgical treatment, blood transfusion, or hospital admission.

To investigate the factors associated with prolonged hemostasis time, a multivariable logistic regression model was constructed (Table 3). The model demonstrated a strong association between the new protocol and shorter hemostasis time [odds ratio (OR) 0.01; *p* < 0.0001] after adjusting for several covariates. The use of antiplatelet therapies was also significantly associated with prolonged hemostasis (OR 1.65 and 2.55, *p* = 0.01 and *p* < 0.001, for single and dual antiplatelet therapy, respectively), whereas the history of hypertension was rather preventive for both outcomes.

**Table 3:** Odds for prolonged hemostasis time (> 180 min)

	Odds ratio	95% CI	*P* value
New protocol	0.01	0.00–0.04	< 0.001
Male	0.80	0.51–1.27	0.35
Age/year	1.01	0.99–1.03	0.38
Sheath size ≥ 5 Fr	0.48	0.20–1.17	0.11
Platelet//mm^3^	0.99	0.97–1.02	0.44
Creatinine/mg/dL	1.30	0.80–2.13	0.29
Hypertension	0.87	0.63–1.19	0.38
Diabetes	0.94	0.70–1.26	0.66
Previous cath/times	1.00	0.95–1.05	0.91
SAPT	1.72	1.15–2.56	0.01
DAPT	2.73	1.80–4.11	< 0.001
DOAC	1.84	0.99–3.38	0.05
Warfarin	1.00	0.48–2.06	0.99
Systolic BP/mmHg	1.00	0.99–1.01	0.88
Body surface area/m^2^	2.50	0.59–10.60	0.18
BMI/kg/m^2^	0.69	0.45–1.03	0.07
Operator: fellows (vs. residents)	1.04	0.70–1.56	0.84
Operator: attendings (vs. residents)	0.69	0.45–1.03	0.07

## Discussion

TRA is the standard and most common approach for coronary angiography today. However, an evidence-based radial compression protocol is still lacking. In this relatively large cohort study, we demonstrated that our new protocol was significantly and strongly associated with (1) a lower incidence of RAO (the primary endpoint) and (2) a shorter hemostasis time (the co-primary endpoint) compared with the traditional commercially recommended radial compression protocol. The effect size of this new protocol was large, suggesting a potentially large clinical benefit for patients.

In the past three decades, studies have shown the advantage of TRA over the traditional trans-femoral approach, mainly in terms of the incidence of complications [11]. TRA causes fewer puncture site complications and requires less restriction of body movement after the procedure [21]. However, the traditional radial compression protocol still takes 2–3 h and sometimes causes RAO, which remains the most frequent complication of TRA [12]. Since it restricts the use of the artery for future procedures, not only catheter angiography but also as a conduit for coronary artery bypass grafting or arteriovenous fistula for hemodialysis, it is important to prevent RAO regardless of whether it is symptomatic or asymptomatic.

One reported mechanism of RAO is acute arterial thrombosis caused by arterial wall injury, some of which resolves later, while others remain occluded [22]. Another mechanism is intimal-medial thickening resulting from vascular injury [23, 24]. Since excessive pressure during hemostasis can damage the arterial wall, compression for hemostasis should be performed with appropriate pressure for as short a time as possible.

Similar protocols were proposed by several doctors including Dr. Ivo Bernat [12]. In this study, we further simplified their methods without using pulse oximeter. Future studies should investigate the non-inferiority and cost-effectiveness of our protocol compared to theirs. The RAO International Group published a consensus paper in 2019, focusing on the incidence, risk factors, and prevention of RAO [12]. In the paper, the group recommends ‘non-occlusive’ or ‘patent’ hemostasis, as well as short compression, since complete occlusion of the artery is a risk for RAO [25]. As stated in the paper, the suggested method using an oximetry-plethysmography device requires a significant work burden. In contrast, the new protocol does not require a special device, although the amount of air is always just before bleeding occurs, and thus the artery is likely to be kept patent. As such, shortened hemostasis time and possibly lower band pressure were the possible mechanisms that reduced the incidence of RAO. Our results also showed that larger body surface area was associated with a lower incidence of RAO regardless of the protocols in line with a previous report [3]. BSA and BMI showed discordant odds against prolonged hemostatic time. One of the underlying mechanisms could be that we usually decided our intravenous heparin doses based on body weight. Another possible mechanism is that a higher BMI, but not BSA, may be associated with thicker wrists and more subcutaneous fat, making hemostasis more difficult. Hypertension and DAPT rates were significantly higher in the conventional protocol group. Both may contribute to longer hemostatic times. Intriguingly, the history of hypertension was associated with shorter hemostasis time and lower rate of RAO even after adjusted by the protocols. Previous studies revealed that the history of hypertension was associated with hyper-coagulant status [26, 27], which might be associated with faster clot formation and thus with fewer RAOs. Further studies are warranted to reassure this association. The number of previous catheter examinations was strongly associated with the risk of RAO. It is not surprising that recurrent injuries of the artery are the risk of RAO, and thus recurrent punctures may be similarly one of the risks. Physicians should be aware of this point and should try to avoid unnecessary punctures of the radial artery. We speculate that larger body size is associated with larger artery diameter [28] and artery-to-sheath ratio which is associated with the incidence of RAO [29], however, further study is needed since we do not have artery diameter information on the present population.

Another important clinical implication of the new protocol is a significant reduction in hemostasis time. Invasive catheter angiography is the gold standard for evaluating anatomical stenosis of the coronary artery and is often performed in an outpatient setting. However, the long resting time after the procedure sometimes prevents patients from returning home on the same day. Outpatient invasive catheter procedures are easier and more accessible if the resting time after the procedure can be shortened. Since the new protocol requires air removal every 30 min, the number of visits to patients may increase in the new protocol. However, a nurse generally has to stay in the patient recovery room until the hemostasis is completed to watch them. Therefore, a short resting time will reduce nurses’ workload and lead to more cost-effective hospital management.

Recently, the distal radial artery approach has been reported as a new puncture method for coronary angiography [30, 31]. This technique may also enable a short-time hemostasis and lower rates of RAO [30, 31]. However, this technique is relatively difficult and cannot be applied to all patients. Besides, a couple of unique complications, such as scaphoid fracture due to injury of the feeding arteries, have been reported [32]. Thus, further evaluation is necessary before the distal radial artery becomes the standard method.

### Limitations

Our study results are best understood in the context of several limitations. First, this is an observational study in which we evaluated the clinical work performed in our hospital. Although we employed statistical analysis to mitigate the risk of confounding and the observed effect size was large, our findings need to be validated in randomized control studies, which should be ideally double-blind. Also, the status of side return of the radial artery should be investigated in future studies. Second, there were a significant number of patients for whom the 6-month radial artery patency data were unavailable, even though over 1300 patients were finally evaluated. Third, we did not routinely perform ultrasound examinations to check for RAO. There may have been some patients whose radial arteries were actually patent. However, even with ultrasound-visible blood flow, an artery without detectable palpitation may still not be feasible for TRA. The frequency of ultrasound examination in each protocol group was low (< 2%) but was not accurately assessed because of the retrospective nature of the study. Although this is a limitation in this study, the impact of this limitation should not be great since most patients were diagnosed with physical examinations and the great difference observed between the groups is not likely to be caused by the small difference in the frequency of ultrasound examinations. Fourth, we did not routinely assess the radial artery diameter using ultrasound. Fifth, the incidence of minor hematoma that does not require surgical treatment, blood transfusion, or hospital stay was not recorded. Sixth, since the new protocol was employed more recently, there might be a technical improvement in the puncture or hemostasis procedures. However, we had performed transradial catheter intervention using the same device for many years before starting this study, and it is likely that the technical learning curves had already achieved a plateau. Sixth, the present study was limited to diagnostic cardiac catheterization, and we dominantly used a 4 Fr sheath. Although the use of a thicker sheath was not associated with prolonged hemostasis time in this study (Table 3), this point should be appropriately acknowledged. Finally, the entire study population were Asian adults, whose body size and body mass index are substantially smaller than those in North America and Europe. Hence, the results may need to be validated in non-Asian countries. Finally, the ACT value might influence the results. However, we did not measure it in this study because it is not cost-effective and practical for routine clinical practice.

## Conclusions

The new radial compression protocol for hemostasis was strongly associated with a shorter hemostasis time and a lower rate of radial artery occlusion in radial-approach coronary angiography. This approach decreases the post-procedural bed rest time, resulting in even fewer complication rates.

## Abbreviations

OR: Odds ratio
RAO: Radial artery occlusion
TRA: Trans-radial approach
